# Secondary insults prevalence, co-occurrence and relationship with outcome after severe TBI

**DOI:** 10.1016/j.bas.2024.102764

**Published:** 2024-02-29

**Authors:** Joseph Donnelly, Erta Beqiri, Frederick A. Zeiler, Peter Smielewski, Marek Czosnyka

**Affiliations:** a Brain Physics Laboratory Division of Neurosurgery Department of Clinical Neurosciences, University of Cambridge, UK; b Department of Neurology, Auckland City Hospital, New Zealand; c Manaaki Manawa—The Centre for Heart Research, University of Auckland, Auckland, New Zealand; d Section of Neurosurgery, Department of Surgery, Rady Faculty of Health Sciences, University of Manitoba, Canada; e Department of Anatomy and Cell Science, Rady Faculty of Health Sciences, University of Manitoba, Canada; f Department of Biomedical Engineering, Price Faculty of Engineering, University of Manitoba, Canada; g Division of Anaesthesia, Department of Medicine, Addenbrooke’s Hospital, University of Cambridge, UK; h Department of Clinical Neuroscience, Karolinska Institutet, Stockholm, Sweden

**Keywords:** PRx, CPP, ICP, Cerebral autoregulation, Traumatic brain injury, Secondary injury

## Abstract

**Introduction:**

Secondary insults due to high intracranial pressure (ICP), low cerebral perfusion pressure (CPP) and impaired cerebral pressure reactivity (PRx) predict outcome after severe traumatic brain injury (TBI).

**Research question:**

What is the prevalence, co-occurrence and prognostic importance of secondary insults due to deranged ICP, CPP or PRx after TBI.

**Material and methods:**

Severe TBI patients requiring ICP monitoring were included. Secondary insults due to ICP, PRx, and CPP were defined as having at least 1 h with a mean value above (or below for CPP) a respective threshold (ICP 20, CPP 60, and PRx 0.25). Percentage time with isolated or co-occurring insults was calculated (impaired ICP only, CPP only, PRx only, ICP and PRx, ICP and CPP, CPP and PRx, ICP CPP and PRx). Prognostic importance for mortality was assessed using a logistic regression model.

**Results:**

822 patients were included of which 76% had elevated ICP, 92% had disturbed pressure reactivity and 55% had low CPP for at least an hour. Out of the total 115,459 h, 46,111 (40%) were spent with at least one variable within the defined secondary injury range. Odds ratios for mortality were greater for combined (impaired ICP, CPP and PRx OR 1.17 95%CI 1.09 to 1.28) than isolated insults (impaired ICP only OR 1.01 95%CI 1.00–1.02, impaired CPP only 1.00 95%CI 0.95–1.05).

**Discussion and conclusion:**

ICP and autoregulation insults are common after TBI and often occur independently. Concurrent ICP, CPP and PRx insults portend worse prognosis than when a single variable is deranged.

## Highlights

• The prevalence, co-occurrence and prognostic importance of secondary insults after TBI are not known.
• In 822 patients, we found that secondary insults were very common for PRx (92%) and ICP (76%).
• Low CPP insults were often associated with impaired PRx or ICP, whereas ICP and PRx insults often occurred in isolation.
• Combined insults of CPP, ICP and PRx were more harmful than isolated insults.

## 1 Introduction

Stabilizing cerebral blood flow (CBF) and preventing raised intracranial pressure (ICP) are two important aims of the neurocritical care of severe traumatic brain injury (TBI) (Le Roux, 2013). The stability of CBF will in turn depend on many factors including the carbon dioxide, temperature, arterial blood pressure (ABP), ICP, and cerebral perfusion pressure (CPP) (Donnelly et al., 2017b; Willie et al., 2014). Of these factors, the stability of CBF in the face of changes in CPP - cerebral autoregulation - is often disturbed after TBI (Armstead et al., 2010; Engelborghs et al., 2000). This, combined with current limitations of continuous CBF monitoring means that CPP monitoring (and therapy) forms a core component of care after severe TBI (Carney et al., 2016; Meyfroidt et al., 2022). ICP is also often monitored after TBI on the basis that it is increased after experimental TBI (Rooker et al., 2003), it contributes to decreased CPP and can serve as warning for evolving intracranial pathophysiology (Hutchinson et al., 2013). Whereas ICP and CPP monitoring are widely implemented after severe TBI, cerebral autoregulation monitoring has been predominantly restricted to clinical research settings. Cerebrovascular pressure reactivity (PRx) provides information about cerebral autoregulation and can be calculated as the moving correlation between 30 consecutive 10-s averages of ABP and ICP.

The combination of continuous ICP, CPP and PRx monitoring therefore has the possibility of allowing a detailed overview of intracranial physiology (Donnelly et al., 2016, 2017a). Despite the theoretical rationale for such monitoring in TBI, several issues will need to be clarified to support a more widespread implementation. First, the proportion of patients that experience impairment in ICP, CPP or PRx has not been established. Second, the co-occurrence of insults due to impaired ICP, CPP or PRx is unknown. Finally, whether incorporating knowledge of cerebrovascular pressure reactivity to the more traditional ICP and CPP monitoring data adds useful information on the patients' physiological state is unclear.

In this descriptive study we focus on secondary injuries due to high ICP, low CPP, and impaired PRx describing their 1) prevalence after severe TBI 2) co-occurrence, and 3) prognostic importance.

## 2 Methods

### 2.1 Patients

822 severe TBI patients entering the neurocritical care unit (NCCU) at Addenbrookes Hospital with ICP monitoring (September 1996–January 2017) were selected. Regional ethical approval was obtained (REC 23/YH/0085). Patients were managed according to TBI guidelines (Menon, 1999) aimed at keeping ICP < 20 mm Hg and CPP > 50–60 mm Hg. CPPopt or PRx-guided management was not part of the management algorithm, although bedside PRx was available to clinicians and could be used at their discretion. Glasgow outcome scale (GOS) score was obtained at 6 months by outpatient assessment, with the following categories: good recovery, moderate disability, severe disability, vegetative state, death (Jennett and Bond, 1975). Unfavourable outcome was defined as severe disability, vegetative state, or death.

### 2.2 Data acquisition and processing

ICP was monitored with an intraparenchymal sensor (Codman ICP Micro- Sensor, Codman & Shurtleff, Raynham, MA). Arterial blood pressure (ABP) was zeroed at the level of the right atrium before 2015 and at the level of the external acoustic meatus thereafter (Baxter Health-care CA, USA; Sidcup, UK). Data were sampled at 100 Hz with proprietary data acquisition and analysis software (ICM+©, https://icmplus.neurosurg.cam.ac.uk/). ABP and ICP signals were cleaned both manually and automatically using simple thresholding criteria for ranges of values and amplitudes. ABP and ICP signals were first averaged (mean) over a 10-s window then PRx was calculated as the moving Pearson correlation of 30 consecutive ABP and ICP, updated every minute.

Hourly averages of ICP, PRx and CPP were calculated. The incidence of significant secondary insults due to ICP, PRx, and CPP were defined as having at least 1 h with a mean value above (or below for CPP) the respective threshold (ICP 20, CPP 60, and PRx 0.25). These ICP and CPP thresholds were chosen as they were the thresholds recommended in the brain trauma foundation guidelines, whereas the PRx threshold was used as this was found to be a threshold with good discrimination between fatal and non-fatal outcomes (Carney et al., 2016; Sorrentino et al., 2012). The co-occurrence of insults due to ICP, CPP and PRx were visualized using a Euler diagram depicting the hours in each of the 7 states possible from the intersection of these three variables (impaired ICP only, impaired CPP only, impaired PRx only, impaired ICP and PRx, impaired ICP and CPP, impaired CPP and PRx, impaired ICP CPP and PRx).

### 2.3 Statistical analysis

Data are reported as means and standard deviations. Univariate generalised linear models were used to assess the influence on outcome of %time spent in each of the 7 states of the euler diagram. We used the R language and software environment for statistical computation (R Core Team, 2015 version 2.12.1) (Team, 2015) using the following packages: eulerr (Larsson, 2016) dplyr (Wickham and Francois, 2016), ggplot2 (Wickham, 2009). The significance level was set at 0.05.

## 3 Results

822 patients were included in the current analysis with a mean age of 39 (Table 1). 643 were male and the majority 598 were severely injured on initial GCS assessment. The remainder had a secondary neurological deterioration. 467 ended up with unfavourable outcome (57%), while 185 (23%) died. The mean duration of monitoring was 131 (standard deviation 109) hours.

**Table 1:** Patient demographics; secondary insults co-occurrence after TBI.

	Overall
n	822
GOS (%)	
Dead	185 (22.5)
Vegetative state	14 (1.7)
Severe disability	268 (32.6)
Moderate disability	204 (24.8)
Good recovery	151 (18.4)
Age [years] (mean (sd))	39.30 (17.18)
Sex = male (%)	643 (78.2)
GCS ≤ 8 (%)	
FALSE	216 (26.3)
TRUE	598 (72.7)
NA	8 (1.0)
Decompressive craniectomy (%)	
FALSE	456 (55.5)
TRUE	183 (22.3)
NA	183 (22.3)
ICP [mm Hg] (mean (sd))	15.13 (7.48)
CPP [mm Hg] (mean (sd))	78.21 (9.06)
PRx [a.u.] (mean (sd))	0.07 (0.17)
Patients with ICP >20 mm Hg > 1 h (n (%))	623 (75.8)
Patients with ICP >40 mm Hg > 1 h (n (%))	93 (11.3)
Patients with CPP <60 mm Hg > 1 h (n (%))	452 (55.0)
Patients with CPP <50 mm Hg > 1 h (n (%))	134 (16.3)
Patients with PRx >0.25 a.u. > 1 h (n (%))	754 (91.7)

*GOS* Glasgow outcome score; *ICP* intracranial pressure; *CPP* cerebral perfusion pressure; *PRx* pressure reactivity index; *NA* Data not available.

Of the 822, 623 (75.79%) had a mean hourly ICP greater than 20 mm Hg for at least 1 h and this percentage was the highest in the group who died (151 (81.62%)). 93 (11.31%) had severe intracranial hypertension (ICP >40 mm Hg for at least an hour). A low CPP (<60 mm Hg) for at least 1 h occurred in 452 (54.99%) patients and occurred the most often in patients who died (n = 123 (66.49%)). The number of patients with sustained (1 h) impaired PRx was 754 (91.73%) and this number was relatively higher in those that died (176 (95.14%)).

**Fig. 1:**
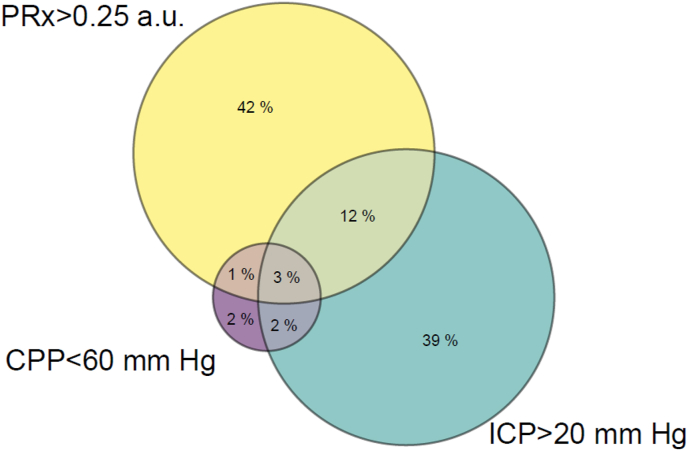
**Euler diagram of hours spent with secondary insults due to high ICP (ICP> 20 mm Hg), low CPP (CPP < 60 mm Hg), and impaired PRx (PRx > 0.25) for entire cohort (115,459 h, n = 822 patients)**. The area of each of the 7 unique sections of the Euler diagram corresponds to the relative number of hours in each adverse physiological condition. Out of the total 115,459 h, 46,111 (40%) were spent with at least one variable within the defined secondary injury range (CPP<60 mm Hg, ICP>20 mm Hg, or PRx>0.25 a.u.). The majority of CPP insults were associated with impaired PRx or ICP. In contrast, the majority of ICP or PRx insults were isolated, i.e. without concomitant derangement in the other 2 physiological parameters. ICP intracranial pressure; CPP cerebral perfusion pressure; PRx pressure reactivity index.

The co-occurrence of the impairments of different combinations of ICP, CPP and PRx across the entire cohort dataset is visualized in a Euler diagram (Fig. 1), in which the relative size of the circle indicates the amount of time (hours) spent in each state of physiological impairment. Out of the total 115,459 h, 46,111 (40%) were spent with at least one variable within the defined secondary injury range (CPP<60 mm Hg, ICP>20 mm Hg, or PRx>0.25 a.u.).

**Fig. 2:**
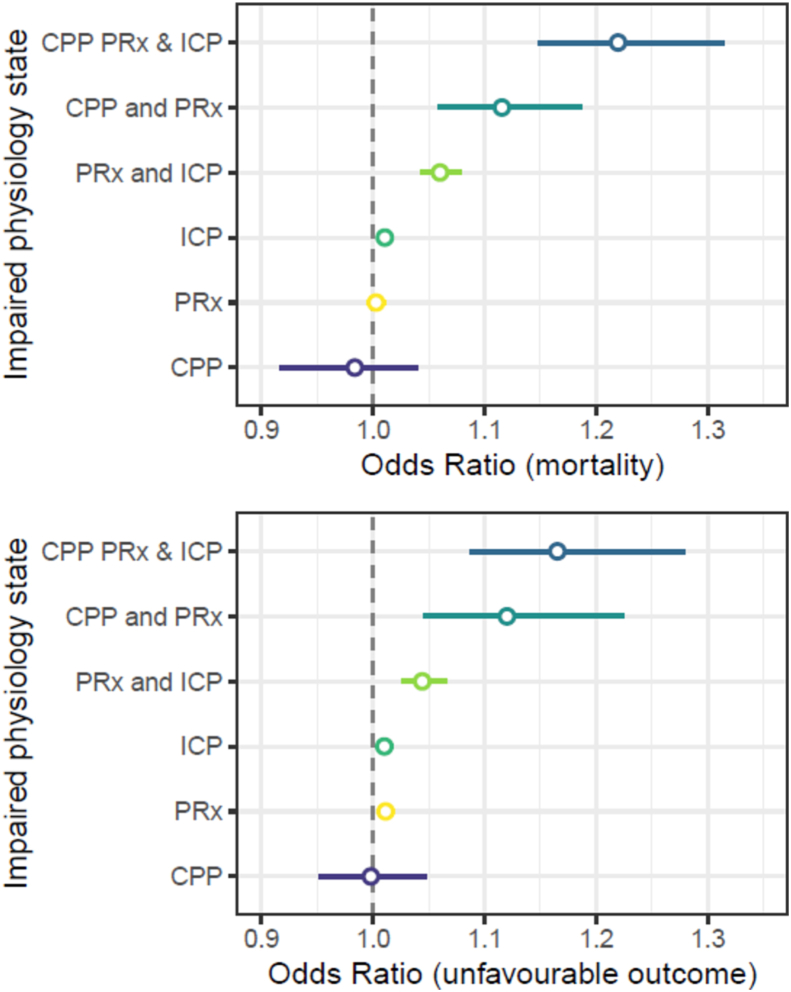
**Odds ratio (95% confidence interval) of percentage time in each impaired physiological state for predicting unfavourable outcome (top) and mortality (bottom)**. In both cases, ICP or CPP insults associated with impaired PRx denoted more extreme odds ratios than ICP insults associated with preserved PRx. This highlights the importance of considering cerebrovascular pressure reactivity (PRx) when interpreting secondary insults due to ICP or CPP. OR odds ratio; PRx pressure reactivity index; ICP intracranial pressure; CPP cerebral perfusion pressure.

To compare the impact of physiological insults in each of the seven states outlined in section 1, univariable odds ratios were constructed for the association of %time in each particular state with patient outcome (mortality or unfavourable outcome). Using this approach, it is clear that states with co-occurring impairments had a stronger relationship (higher odds ratio) with outcome than when only a single facet of physiology was impaired (Fig. 2). For example, odds ratios for mortality were greater for combined (impaired ICP, CPP and PRx OR 1.17 95%CI 1.09 to 1.28) than isolated insults (impaired ICP only OR 1.01 95%CI 1.00–1.02, impaired CPP only 1.00 95%CI 0.95–1.05).

## 4 Discussion

Using a comparatively large severe TBI neuromonitoring database we describe the high incidence of sustained ICP (76%) and PRx (92%) secondary insults and a relatively lower incidence of sustained insults due to CPP (55%). Although some of these insults occurred at the same time, the majority occurred independent of each other. Finally, an ICP or CPP insult was more damaging if it was associated with impaired compared with intact autoregulation. Together these data highlight the pervasiveness of cerebral insults after TBI and the potential added value of autoregulation monitoring in interpreting a ICP or CPP insult.

### 4.1 ICP secondary insults

Sustained ICP insults (greater than 20 mm Hg) occurred in over three-quarters of the cohort despite a protocol targeted at preventing raised ICP. Although the definition of an ICP insult (greater than 20 mm Hg for more 1 h) is somewhat arbitrary, it fits with recent analytical studies indicating that ICP > 20 for an hour is associated with poorer outcome (Güiza et al., 2015), and also with clinical guidelines which advocate treatment when ICP is above ∼ 20 mm Hg (Carney et al., 2016; Meyfroidt et al., 2022). What makes some patients vulnerable to increases in ICP and others more resistant is unclear and likely complex considering that ICP increases can occur with an increase in volume of any of the blood, parenchymal, or CSF compartment after TBI.

### 4.2 CPP secondary insults

CPP insults in this cohort were less common than ICP or PRx insults with just over half of patients having an average CPP <60 mm Hg for at least 1 h. The relatively low (compared to PRx and ICP) prevalence is probably because CPP can be more readily modulated by increasing the dose of vasopressors as directed in the ICP/CPP treatment protocol. Interestingly, low CPP was often (∼three-quarters of the time) associated with an increased ICP or impaired PRx (Fig. 1).

The concept of CPP as the difference between ABP and ICP has had significant implications for TBI management. In TBI, where raised ICP is a prevalent problem, one way of ensuring the brain has an adequate perfusion pressure and thus cerebral blood flow is by pharmacologically increasing ABP. This led to the CPP-oriented protocols of Rosner where CPP was increased to relatively high values (Rosner et al., 1995). Vigorous CPP targeting with vasopressors can however have negative effects with myocardial and pulmonary dysfunction common (Robertson et al., 1999).

### 4.3 Pressure reactivity secondary insults

Pressure reactivity impairment was common in this cohort with ∼90% of patients experiencing at least 1 h of impaired PRx (Table 1). As expected, we found that impaired PRx potentiated the effect of a high ICP or a low CPP (Fig. 2). While the combination of impaired PRx and low CPP was rare in this cohort, impaired PRx with normal CPP was much more common and could represent a few scenarios – A rightward shift in the ‘autoregulation curve’, elevated carbon dioxide, elevated temperature, a CPP above the upper limit if autoregulation or inherent noise associated with the PRx signal.

The causes and consequences of impaired PRx after TBI are uncertain. The impairment could stem from the injury itself, or could be related to changes in blood gases, temperature, or medication (Lavinio et al., 2007; Sekhon et al., 2015). On the other hand, impaired PRx implicates passive transmission of ABP slow waves to ICP which may potentially cause regional microvascular damage and through this mechanism may have a significant effect on patient outcome. Whether the poor prognosis associated with disturbed PRx is due to the transmission of ABP waves to cerebral blood volume *per se*, or a more general reflection of a disturbed cerebrovascular system is unknown.

### 4.4 Combination monitoring

The distinction between combined physiologic insults, and single physiologic insults is important - the odds of mortality or unfavourable outcome were higher if an ICP or CPP insult was associated with impaired PRx. These results are supported by studies utilising the Brain IT database (minute-by-minute data) that found that if estimated cerebral autoregulation was intact (using a lower frequency analogue of PRx), ICP and CPP insults had less of an effect on patient outcome (Ai Åkerlund et al., 2020; Güiza et al., 2015, 2016). Focusing on CPP, ICP and PRx, we found that the majority of insults occurred independently. This was particularly so for ICP and PRx and highlights the fact that although the ICP signal is used in the calculation of PRx, it is measuring a different aspect of physiology.

A potential contributor to the lack of progress in evidence-based treatments after TBI is that in clinical trials, physiologic insults are usually viewed in isolation. However, in the complex case of a severely injured TBI patient, a particular physiological impairment is normally viewed in a broader context. Although the rationale underpinning such a personalised treatment approach is straight-forward, the multiple facets that could or should be considered make the implementation of a truly personalised therapy after TBI difficult. Integrating CPP and PRx with ‘CPPoptimal’ or CPP above the lower limit of reactivity are preliminary steps in this direction (Beqiri et al., 2023; Tas et al., 2021).

### 4.5 Limitations

The denominator for prevalence estimate is the number of TBI patients receiving ICP monitoring and having their ICP and ABP signals recorded, which in turn depends on the local clinician’s decision on whether ICP monitoring is warranted and whether the research team was available to connect the patient to the computerized monitoring system. In addition, due to the nature of clinical monitoring, interruptions in the data stream are inevitable, for example due to transfer of the patient to scanner or operating theatre. In the majority of this cohort (prior to 2015), the ABP sensor was calibrated to zero at the level of the right atrium, while after 2015 this was changed to the level of the external acoustic meatus to align with national guidelines (Thomas et al., 2015). Thus, a single ABP will vary ∼10 mm Hg depending on where the transducer is zeroed (zeroed at the ear reads ∼10 mm Hg lower than at the heart). This could also have contributed to the relatively low number of hours spent with a CPP below 60 mm Hg in this cohort. Information on therapeutic intensity, or indeed withdrawal of therapy was not available in this retrospective analysis and thus relationships between impaired ICP and mortality may be heavily influenced by small number of cases where high ICP was monitored after withdrawal of therapy.

### 4.6 Conclusions

ICP and autoregulation insults are common (76% and 92% respectively) after TBI and often occur independently. Concurrent ICP, CPP and PRx insults portend worse prognosis than when a single variable is deranged. Together these data point to the potential benefit of integrating intracranial monitoring modalities at the bedside after severe TBI.

## Funding

Erta Beqiri is supported by the 10.13039/501100007155Medical Research Council (grant no.: MR N013433-1) and by the Gates Cambridge Scholarship. FAZ receives research support from the 10.13039/100010318University of Manitoba - Manitoba Public Insurance (MPI) Professorship in Neuroscience, the 10.13039/501100000038Natural Sciences and Engineering Research Council of Canada (NSERC) (DGECR-2022-00260, RGPIN-2022-03621, ALLRP-578524-22, and ALLRP-576386-22), 10.13039/501100000024Canadian Institutes of Health Research (CIHR) (Grant #: 472286), the MPI Neuroscience Research Operating Fund, the 10.13039/100008252Health Sciences Centre Foundation Winnipeg, the 10.13039/501100000196Canada Foundation for Innovation (CFI) (Project #: 38583), and 10.13039/100008794Research Manitoba (Grant #: 3906, 5429).

## Declaration of competing interest

The authors declare the following financial interests/personal relationships which may be considered as potential competing interests:Peter Smielewski reports financial support was provided by ERDF (European Regional Development Fund), Interreg France (Channel) England Programme. Peter Smielewski reports a relationship with Cambridge Enterprise Ltd, Cambridge, U.K. that includes: consulting or advisory. Peter Smielewski has patent with royalties paid to Cambridge Enterprise Ltd, Cambridge, U.K. Marek Czosnyka reports a relationship with Cambridge Enterprise Ltd, Cambridge, U.K. that includes: consulting or advisory. Marek Czosnyka has patent with royalties paid to Cambridge Enterprise Ltd, Cambridge, U.K.
